# Genomic Analysis of Natural Rough *Brucella* *melitensis* Rev.1 Vaccine Strains: Identification and Characterization of Mutations in Key Genes Associated with Bacterial LPS Biosynthesis and Virulence

**DOI:** 10.3390/ijms21249341

**Published:** 2020-12-08

**Authors:** David Kornspan, Regina Lubkovskaia, Shubham Mathur, Adva Yeheskel, Mali Salmon-Divon

**Affiliations:** 1 Department of Bacteriology, Kimron Veterinary Institute, Bet Dagan 50250, Israel; r.lubkovskaia@gmail.com (R.L.); mathur.shubham1990@gmail.com (S.M.); 2 Genomic Bioinformatics Laboratory, Department of Molecular Biology, Ariel University, Ariel 40700, Israel; malisa@ariel.ac.il; 3 School of Public Health, Sackler Faculty of Medicine, Tel Aviv University, Tel Aviv 6997801, Israel; 4 Bioinformatics Unit, George S. Wise Faculty of Life Sciences, Tel Aviv University, Tel Aviv 6997801, Israel; suezadva@gmail.com; 5 Adelson School of Medicine, Ariel University, Ariel 40700, Israel

**Keywords:** *Brucella melitensis* Rev.1, vaccine, comparative genomic analysis, LPS genetics, virulence attenuation

## Abstract

*Brucella* species are facultative intracellular bacteria that cause brucellosis, a zoonotic world-wide disease. The live attenuated *B. melitensis* Rev.1 vaccine strain is widely used for the control of brucellosis in the small ruminant population. However, Rev.1 induces antibodies against the O-polysaccharide (O-PS) of the smooth lipopolysaccharide thus, it is difficult to differentiate between infected and vaccinated animals. Hence, rough *Brucella* strains lacking the O-PS have been introduced. In the current study, we conducted a comprehensive comparative analysis of the genome sequence of two natural Rev.1 rough strains, isolated from sheep, against that of 24 Rev.1 smooth strains and the virulent reference strain *B. melitensis* 16M. We identified and characterized eight vital mutations within highly important genes associated with *Brucella* lipopolysaccharide (LPS) biosynthesis and virulence, which may explain the mechanisms underlying the formation of the Rev.1 rough phenotype and may be used to determine the mechanism underlying virulence attenuation. Further complementation studies aimed to estimate the specific role of these mutations in affecting *Brucella* morphology and virulence will serve as a basis for the design of new attenuated vaccines for animal immunization against brucellosis.

## 1. Introduction

*Brucella* species are Gram-negative, facultative intracellular bacteria that cause brucellosis, a zoonotic world-wide disease [1]. As in other Gram-negative bacteria, its outer membrane plays a crucial role in the infection process, being the first point of bacterial interaction with its host [2]. The major component of the outer membrane, the lipopolysaccharide (LPS), consists of three structural regions: (1) The lipid A, which forms the hydrophobic anchor of the LPS within the outer membrane and is responsible for many endotoxic properties attributed to LPS, (2) an inner and outer core composed of sugar molecules, and (3) the O-antigen (O-polysaccharide; O-PS) a polymerized sugar chain extending into the extracellular environment [3]. Most *Brucella* species occur naturally as smooth LPS (S-LPS) strains (i.e., *Brucella abortus, B. melitensis, B. suis, B. neotomae, B. microti, B. pinnipedialis*, and *B. ceti*), which contain LPS that is modified with an O-antigen, while *B. ovis, B. canis, B. abortus* RB51, and *B. melitensis* B115 are typically rough strains (R-LPS) which lack the O-side chain from LPS [3,4]. Generally, rough strains were shown to be less virulent than smooth strains, with the exception of *Brucella ovis* and *B. canis*, which are rough but virulent [5]. Indeed, mutants of *B. melitensis, B. suis*, and *B. abortus* lacking the O-antigen modification were shown to be considerably less virulent than their respective parent strains [6,7,8]. It has been suggested, that the *Brucella* LPS plays a key role in the invasion, intracellular multiplication and protection against complement-mediated lysis [3]. *Brucella* LPS is a significantly less effective inducer of the innate immunity compared to that of *Escherichia coli*. As such, *Brucella* is able to evade detection by the host immune system and to form a chronic intracellular infection [9,10].

*B. melitensis*, which infects sheep and goats mainly in Africa, the Middle East, Asia, and Latin America, is one of the most pathogenic *Brucella* species for humans [11]. In these high-prevalence regions, brucellosis is controlled mainly by vaccination of the host ruminants. Among the widely used brucellosis vaccines is the live attenuated Rev.1 *B. melitensis* strain [12] which successfully protects and reduces abortions in small ruminants [13,14]. However, Rev.1 has some undesirable traits: It may be abortifacient when used in pregnant animals, it is infectious for humans, and it induces O-PS-specific antibodies, which interfere with the serological tests that employ S-LPS as an antigen [5,14]. As compared with the *B. melitensis* Rev.1 smooth strain, rough *B. melitensis* strains may offer a solution to these drawbacks, as they are less virulent and lack O-PS [6,15]. Because of these features, rough *Brucella* mutants have been considered potential brucellosis vaccines [14,16].

We have recently used RNA-seq to comprehensively compare the transcriptome of smooth Rev.1 vaccine strains to that of natural rough Rev.1 vaccine strains, each grown under either normal or acidic conditions, in order to better understand the molecular mechanisms underlying the virulence attenuation of the rough versus smooth Rev.1 strains [17]. We found 18 key genes that were significantly differentially expressed between smooth and rough strains, all associated with bacterial virulence and survival [17]. We also showed, in a cell culture model, that the rough Rev.1 strain was highly attenuated compared to the smooth Rev.1 strain [17].

In this study, we evaluated the genetic characteristics of two natural Rev.1 rough strains, isolated from sheep, compared to 24 Rev.1 smooth strains (including the original Elberg *B. melitensis* Rev.1 vaccine strain) and the virulent smooth reference *B. melitensis* 16M strain, in order to reveal the specific gene mutations in the genomes of the natural Rev.1 rough strains. We identified and characterized several vital mutations within highly important genes associated with *Brucella* LPS biosynthesis and virulence, which may explain the mechanisms underlying the formation of the Rev.1 rough phenotype, thereby improving our understanding of the genetic structure relationship in *Brucella* LPS, and revealing new insights into the virulence attenuation of these strains.

## 2. Results and Discussion

Genome-wide comparisons between two natural rough *B. melitensis* Rev.1 vaccine strains (71036 and 44457), 24 smooth *B. melitensis* Rev.1 vaccine strains and the *B. melitensis* 16M virulent strain were conducted to identify all the single-nucleotide polymorphisms (SNPs) between these genomes. Below, we first report the genes containing unique SNPs in the natural rough Rev.1 vaccine strains compared to their smooth strain counterparts and to the virulent 16M smooth strain. We then characterize each mutation and evaluate their potential effect on protein function in order to better understand the molecular mechanisms leading to the formation of the Rev.1 rough phenotype and to the virulence attenuation of these strains. 

### 2.1. Genomic SNPs

**Figure 1:**
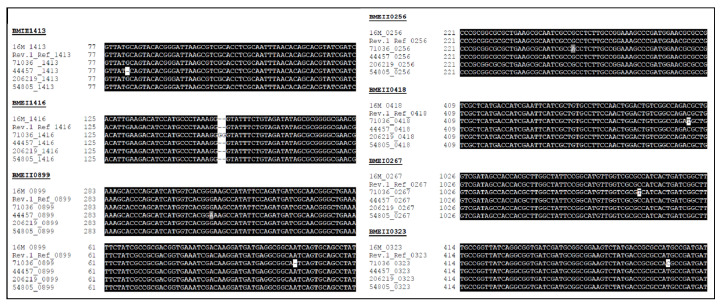
Sanger validation of selected variants. Black color shows conserved regions, gray and white color represent mutant regions.

The genome-wide comparisons between the natural rough *B. melitensis* Rev.1 vaccine strain, 71036, and the original Elberg smooth Rev.1 vaccine strain revealed a total of 12 variants (Supplementary Table S2) of which seven exclusively appeared in this strain and not in other tested Rev.1 smooth strains. Among these, one SNP is a synonymous substitution encoding the same amino acid; four SNPs are missense substitutions encoding a different amino acid; and the remaining two SNPs contain frameshift mutations, which, in many cases, have a deleterious effect on the protein function. A comparison between the natural rough *B. melitensis* Rev.1 vaccine strain 44457 and the original Elberg smooth Rev.1 vaccine strain revealed a total of 12 variations of which three exclusively appeared in this strain and not in other tested Rev.1 smooth strains. Among these, one is located in intergenic regions; one is a synonymous substitution; and one is a missense substitution. We next compared the two natural rough *B. melitensis* Rev.1 vaccine strains, 71036 and 44457, and the *B. melitensis* 16M virulent strain and found two additional unique SNPs in 44457 that did not appear in our tested Rev.1 smooth strains. One SNP is an upstream gene variant, and another is a frameshift variant (Supplementary Table S2, rows 21–22). We assume that these SNPs were not detected in our comparison with the Rev.1 sequence due to incomplete sequencing or annotation of the Rev.1 reference genome. Only a single gene, BMEII0899, was mutated both in 71036 and 44457 rough strains, albeit with different unique mutations. To validate the existence of the detected rough-unique mutations, we applied Sanger sequencing of 8 detected variants to the two Rev.1 rough strains and three Rev.1 smooth strains including the original Elberg smooth Rev.1 vaccine strain. All the variants selected for Sanger sequencing were validated (Figure 1).

### 2.2. Characterization of the Specific SNPs of the Natural Rough B. melitensis Rev.1 71036 Vaccine Strain

#### 2.2.1. Genes Involved in LPS Biosynthesis

##### Phosphomannomutase (BMEII0899)

We identified a frameshift mutation (c.388delA|p.Ile130fs) in the CUC12_12640 (BMEII0899) gene encoding phosphomannomutase. Phosphomannomutase is encoded by the *B. melitensis* manBcore gene which is responsible for the interconversion of mannose-6-phosphate to mannose-1-phosphate [5]. Phosphomannomutase enzymes contain four domains with the active site residues located between these domains [18]. Each domain contributes to the enzyme function in a different way: Phosphoglucomutase_phosphomannomutase (PGM_PMM)_I contains the catalytic phosphoserine, PGM_PMM_II binds the magnesium ion which is important for enzymatic activity, PGM_PMM_III recognizes the sugar orientations, and PGM_PMM_IV binds the phosphate group [19]. This frameshift mutation will delete three of the PGM-PMM domains, and thereby most probably cause a non-functional protein. In *Brucella*, mannose acts as an important precursor in the O-antigen biosynthetic pathway and in the production of the inner core moiety of LPS [5]. Using transposon mutagenesis in *B. melitensis* H38 and in *B. abortus* 2308, it has been shown over a decade ago that mutations in this gene resulted in the *Brucella* rough phenotype [7,15]. The *B. melitensis* H38 manBcore mutant was unable to grow in bone-marrow derived macrophages (BMDM), and was completely degraded in LAMP1-positive compartments [15]. We assume that the detected frameshift mutation may not only highly affect the conversion of smooth to rough phenotype in the Rev.1 rough strain, but also the survival capabilities of this microorganism within its host.

##### ABC Transporter Permease (BMEI1416)

We identified a frameshift mutation (c.151_152dupGG|p.Ile52fs) in the *wzt* (CUC12_10065, BMEI1416) gene encoding ABC transporter permease. The mutation is located on the ABC_trans domain (PFAM PF00005; residues 49–182). The frameshift mutation is predicted to cause a non-functional protein due to the missing domain. 

The O-antigen homopolymers of *Brucella* are fully synthesized and assembled at the cytoplasmic face of the inner membrane and then transported across the inner membrane using an ATP-binding cassette (ABC) transporter system [2,20]. In *B. melitensis*, the genes *wzm* (BMEI1415) and *wzt* (BMEI1416) were predicted to form the transmembrane and ATPase domain, respectively, of an ABC transporter required for the translocation of the full length homopolymer O-antigen, from the cytoplasmic to the periplasmic face of the inner membrane [2,20]. Indeed, the deletion of *wzm*/*wzt* resulted in a rough phenotype of *B. melitensis* 16M colonies and in the accumulation of intracellular O-antigen [20]. The wild type smooth phenotype was restored via mutant complementation experiments, demonstrating the high significance of the transmembrane and ATPase domain in the formation of the *Brucella* smooth phenotype [20]. In contrast, in the natural rough *B. melitensis* B115 strain, the same complementation failed to restore the smooth phenotype, indicating that additional mutations may be present in this strain that affect the LPS O-side chain transport or ligation to the lipid A-core molecule [20]. We assume that the detected frameshift mutation may highly affect the conversion of smooth to rough phenotype in this Rev.1 rough strain.

#### 2.2.2. Genes Involved in Survival within Environmental Stress Conditions

##### Glutathione S-Transferase Family Protein (BMEII0256)

**Figure 2:**
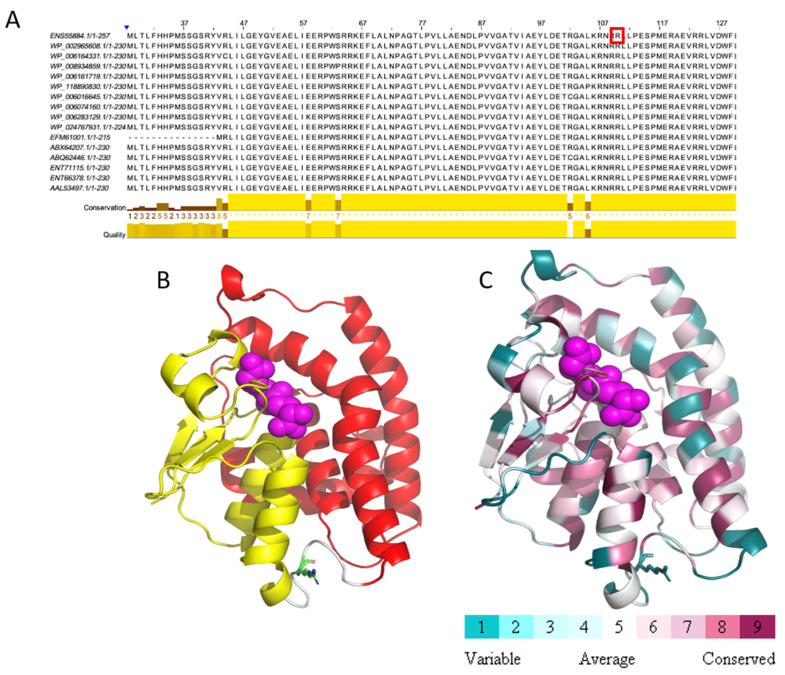
Missense mutation within glutathione S-transferase protein. (**A**) MSA of 16 *Brucella* glutathione S-transferase sequences. Position 83, marked by red rectangle, is conserved as arginine. (**B**) Prosite motifs are shown on the model structure of glutathione S-transferase (GST-C TER in red, GST-N TER in yellow). Glutathione in magenta spheres, Arg83 in green sticks. (**C**) Glutathione S-transferase is colored according to conservation. Glutathione in magenta spheres, Arg83 in sticks. This analysis is based on 150 homologous sequences from Uniref90.

We identified a missense mutation (c.248G>A) in the CUC12_15955 (BMEII0256) gene encoding a glutathione S-transferase family protein (GST). This mutation leads to the replacement of the amino acid arginine to histidine at position 83 of the protein sequence. This mutation is unique, as when we compared the GST sequences among 16 *Brucella* species/strains, we found that Arg83 was conserved through all homologs (Figure 2A). To evaluate the possible effect of the detected mutation on protein functionality, we conducted a structural analysis of the amino acid sequence of GST based on the solved 3D structure of this protein from *Sinorhizobium meliloti* (PDB ID 4MDC). The missense mutation at position 83 is located within the GST_N_3 domain (residues 4–86), which contain two Prosite motifs: GST-C TER and GST-N TER, between residues 1–79 and 88–222, respectively (Figure 2B,C). This mutation is predicted to have either an allosteric effect on the catalytic function or a local structural effect due to histidine being a smaller residue than arginine. 

*Brucella* is a facultative intracellular pathogen, which is able to survive and replicate within various types of host cells [21,22,23]. Therefore, it has to adapt to a range of different hostile environments, including oxidative and acid pH stresses [24]. GSTs are evolutionarily conserved enzymes which play an active role in the detoxification of various xenobiotic compounds and protect cells from oxidative stress by detoxifying some of the secondary reactive oxygen species (ROS), including hydrogen peroxide, hydroxyl radicals, and superoxide anions [25,26]. Indeed, a glutathione S-transferase B1-1 null mutant of *Proteus mirabilis* was found to be more sensitive to oxidative stress than its wild-type counterpart [27]. Accordingly, it was suggested that this GST plays an important role in protecting against oxidative stress [27]. We assume that the detected missense mutation may affect the GST function, resulting in a lower capability to cope with oxidative stress thereby contributing to the attenuation of this strain.

##### NAD(P) Transhydrogenase Subunit Alpha (BMEII0323)

We identified a missense mutation (c.464T>C) in the CUC12_15600 (BMEII0323) gene encoding NAD(P) transhydrogenase subunit alpha. This mutation leads to the replacement of the amino acid methionine to threonine at position 155 of the protein sequence. The NAD(P) transhydrogenase subunit alpha protein is very conserved in *Brucella* and in other Gram-negative bacteria (Supplementary Figure S1A), and specifically high conservation is seen in this methionine residue. NAD(P) transhydrogenase is a tetramer composed of two alpha and two beta subunits. This protein does not have a crystal structure and its 3D structure was modeled using Alpha1 dimer of *Thermus thermophilus* transhydrogenase (PDB ID 4IZH, article is unpublished). The detected mutation is located in the alpha subunit of the enzyme. A Prosite motif was detected within this protein, between residues 172–197: ALADH_PNT_2, an alanine dehydrogenase and pyridine nucleotide transhydrogenase signature 2 pattern (Prosite pattern ID PS00837; Supplementary Figure S1B). Although Met155 is highly conserved and is located in a conserved region of the protein (Supplementary Figure S1C), it does not interact with the NADH substrate of the enzyme and is not located within the specific detected Prosite pattern, and so its role is predicted to be mainly structural. Mutation to threonine, although a polar residue and of a smaller size, is hypothesized to have a local structural effect.

NAD(P) transhydrogenase, an integral membrane protein found in most organisms, catalyzes the transfer of reducing equivalents between NAD(H) and NADP(H), coupled to the translocation of protons across a membrane [28]. The resulting NADPH is subsequently used for biosynthetic reactions or for the reduction of glutathione (GSH) [28]. Indeed, the highly essential enzyme, NADPH-dependent glutathione reductase, is known to recycle oxidized glutathione (glutathione disulfide; GSSG) produced during bacterial growth and under oxidative stress, back to the reduced form (GSH) [29]. It is possible that the detected missense mutation in this rough strain may affect the efficiency of the NADPH production by NAD(P) transhydrogenase, resulting in a lower intracellular GSH/GSSG ratio, reducing the capability of this strain to cope with oxidative stress.

##### Magnesium Transporter (BMEII0418)

We identified a missense mutation (c.464C>T) in the BMEII0418 gene encoding a magnesium (Mg^2+^) transporter. This mutation leads to the replacement of the amino acid threonine to methionine at position 155 of the protein sequence. The mutation is located within the CBS domain (residues 135–197). This domain’s 3D structure was modeled using the magnesium transporter MgtE protein from *Thermus thermophilus* (PDB ID 2ZY9, Supplementary Figure S2B–D) [30]. Thr155 is located in a conserved hydrophobic core of the CBS motif. The mutation is predicted to have a structural effect on the motif, since methionine is larger than threonine. Notably, this mutation is unique since, when we compared the sequence of the CBS domain of 85 *Brucella* magnesium transporter sequences, Thr155 was conserved throughout all homologs (Supplementary Figure S2A,D).

Mg^2+^ is the most abundant divalent cation in living cells and the second most abundant cation after K+ [31,32]. Mg^2+^ plays several essential roles, including stabilizing macromolecular complexes and membranes, neutralizing nucleic acids, and nucleotides in the cytoplasm, and acting as a cofactor in a variety of enzymatic reactions [31]. As magnesium is an essential nutrient, bacteria possess multiple transporters for Mg^2+^. In *Salmonella typhimurium*, a subset of Mg^2+^ transporters was shown to be highly induced upon invasion of this bacterium into eukaryotic cells, and the signal for this induction was shown to be Mg^2+^ itself, mediated by the PhoP/PhoQ two component regulatory system, important for virulence, thus implicating Mg^2+^ and its transporters in pathogenesis [33,34]. Furthermore, in *Yersinia pestis* and *Y. pseudotuberculosis*, Mg^2+^ was shown to be an important signal controlling gene regulation via the PhoP/PhoQ two-component regulatory system [35,36,37]. Notably, the *mgtC* gene, first described in *S. Typhimurium* as part of the *mgtCB* operon encoding an Mg^2+^ transporter [38], was shown to play a major role in intramacrophage survival and growth under magnesium limitation in *B. suis* [39]. We assume that the detected missense mutation may lead to the formation of a less functional magnesium transporter, resulting in a lower capability of this rough strain to survive within its host, thereby contributing to the attenuation of this strain.

##### MFS Efflux Pump (BMEI0267)

We identified a missense mutation (c.1069C>T) in the BMEI0267 gene encoding an MFS efflux pump. This mutation leads to the replacement of the amino acid proline to serine at position 357 of the protein sequence. MFS transporters, which are membrane transport proteins that facilitate movement of small solutes across cell membranes in response to chemiosmotic gradients, contain a 12-transmembrane (TM)-helix core consisting of two pseudo-symmetrical six-helix domains [40]. A membrane-embedded central cavity is present between these two domains, thus forming the substrate-transport path. This protein does not have a crystal structure and its 3D structure was modeled using *E. coli* multidrug transporter MdfA (PDB ID 4ZP0, [41]). The detected mutation is located within the MFS Prosite motif (residues 2–395) of the protein. This protein is very conserved in *Brucella* and in other Gram-negative bacteria (Supplementary Figure S3A), and specifically high conservation is seen in this proline residue. Pro357 is located in TM helix 11 (Supplementary Figure S3B) and although it is highly conserved and is located in a very conserved region of the protein (Supplementary Figure S3C), its role is predicted to be mainly structural, as alignment to other bacterial MFS proteins does not show it to have a role in substrate binding (Supplementary Figure S3B). For this reason, mutation to serine, a polar residue, but of similar size to proline, is hypothesized to have a local structural effect.

MFS efflux pumps are membrane protein complexes that are conserved in all living organisms [42,43]. In several pathogenic bacteria (e.g., *Listeria monocytogenes* and *Vibrio cholera*), efflux pumps were shown to be involved in intracellular survival, resistance to stress, antibiotic extrusion and biofilm formation [44,45]. Furthermore, the important role of efflux pumps in the bacterial invasion process and survival within macrophages and intestinal epithelial cells was shown in *S. typhimurium* [46]. We assume that the detected missense mutation of the Rev.1 rough strain may have a negative impact on the invasion capability and survival of this microorganism within its host, thereby contributing to the attenuation of this strain.

### 2.3. Characterization of the Specific SNPs in Genes Involved in LPS Biosynthesis of the Natural Rough B. melitensis Rev.1 44457 Vaccine Strain

#### 2.3.1. Genes Involved in LPS Biosynthesis

##### Phosphomannomutase (BMEII0899)

**Figure 3:**
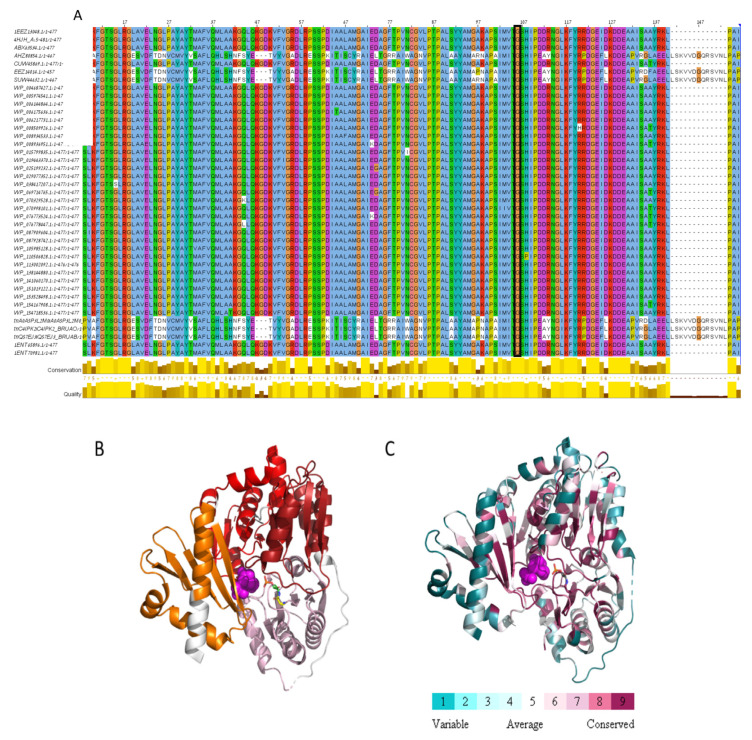
Missense mutation within Phosphomannomutase. (**A**) MSA of 41 *Brucella* Phosphomannomutase sequences. Only the first Phosphoglucomutase-phosphomannomutase (PGM-PMM)_is presented (residues 1–140). Gly103, marked by black rectangle, is conserved through all homologs. (**B**) The protein domains are shown on the model structure of glutathione S-transferase (PGM-PMM-I in pink, PGM-PMM-II in dark red, PGM-PMM-III in red, PGM-PMM-IV in orange). Glucose-6-phosphate in magenta spheres, catalytic phospho-histidine 104 in green carbons, Glycine 103 in yellow carbons. (**C**) PGM/PMM is colored according to conservation. Glucose-6-phosphate in magenta spheres. Positions 103 and 104 in sticks. This analysis is based on 150 homologous sequences from Uniref90.

We identified a missense mutation (c.308G>A) in the BMEII0899 gene encoding phosphomannomutase. This mutation leads to the replacement of the amino acid glycine to glutamic acid at position 103 of the protein sequence. This mutation is unique since, when we compared the phosphomannomutase sequences among 41 *Brucella* species/strains, we found that Gly103 was conserved through all homologs (Figure 3A). To evaluate the possible effect of the detected mutation on protein functionality, we conducted a structural analysis of the amino acid sequence of phosphomannomutase based on the solved 3D structure of its paralog phosphoglucomutase (PDB ID 4HJH, Figure 3B,C). The missense mutation at position 103 of the amino acid sequence, replacing glycine with glutamic acid (two amino acids which are widely different in hydrophobicity, size, and charge), is located within the PGM_PMM_I domain (5–140), and is predicted to cause reduced function since it is highly conserved and is located proximal to the active site residues (residues 9, 14, 114, 299), to the substrate binding site (residues 11, 104, 247, 281, 198, 300, 322, 324, 326, 436, 438, 439, 440, 445) and to the metal binding site (residues 104, 242, 244, 246). As mentioned above, mutations in this gene resulted in the *Brucella* rough phenotype and were shown to be attenuated compared to their parental smooth strains [7,15]. We therefore assume that the detected missense mutation in phosphomannomutase may highly affect the conversion of smooth to rough phenotype in Rev.1 and contribute to the attenuation of this strain.

##### GDP-Mannose Dehydratase (BMEI1413)

We identified a frameshift mutation (c.82delG|p.Ala28fs) in the *gmd* (BMEI1413) gene encoding GDP-mannose dehydratase (GMD). Position 28 of the amino acid sequence of GMD is located within the GDP-mannose dehydrogenase domain (residues 6–336). Therefore, we predict this mutation leads to a lack of a functional domain. 

In *Brucella*, most of the genes involved in LPS biosynthesis are clustered in the *wbk* and *wbo* genetic regions [3,16]. Wbk is the major genetic region of *Brucella* O-PS synthesis, and contains genes encoding for enzymes necessary for N-formylperosamine synthesis (*gmd*, *per*, *wbkC*), two O-PS glycosyltransferases (*wbkE*, *wbkA*), ABC transporters (*wzm*, *wzt*), the polyisoprenyl-phosphate Nacetylhexosamine-1-phosphate transferases (PNPT) enzyme (*wbkF*) and at least one enzyme necessary for the synthesis of an N-acetylaminosugar (*wbkD*) [47]. Based on sequence similarity, the gene products of *gmd* and *per* were assumed to be involved in the biosynthesis and modification of the monomer of the *Brucella* LPS O-side chain, where the GDP-mannose is dehydrated by the GDP-mannose dehydratase, Gmd, to form the GDP-4-keto-6-deoxymannose which is then converted to GDP-4-NH2-4,6-dideoxymannose (GDP-perosamine) by the perosamine synthetase, Per [20]. Mutations in the *gmd* gene resulted in the *Brucella* rough phenotype and these mutants were shown to be attenuated compared to their parental smooth strains [15]. We therefore assume that the detected missense mutation in GMD may not only highly affect the conversion of smooth to rough phenotype in this strain, but may also contribute to the attenuation of this strain.

### 2.4. In Vitro Acidic and Oxidative Stress Survival of the Natural Rough B. melitensis Rev.1 71036 and 44457 Vaccine Strains

As described above, we have detected mutations in the BMEII0256 and BMEII0323 genes of the natural rough *B. melitensis* Rev.1 71036 strain but not in the natural rough *B. melitensis* Rev.1 44457 strain or in the Rev.1 smooth strains. These genes encode proteins that play a vital role in the bacterial response to various stress conditions [25,26,27,29]. We therefore investigated the survival of these strains under acidic and oxidative stress conditions in order to determine the potential association between the detected mutations and bacterial survival under these stress conditions. Notably, when bacteria where grown in an acidic environment (pH 4.4, 3 h) only minor differences in CFU/mL were detected between both natural rough Rev.1 strains (Supplementary Figure S4A). However, when bacteria were grown under oxidative stress conditions (10 mM H_2_O_2_, 2 h), strain 71036 exhibited a decrease of viable CFU/mL, compared to strain 44457 (Supplementary Figure S4B). It therefore appears that the specific mutations in these highly important genes decrease the bacterial ability to cope with oxidative stress in the host, thus contributing to bacterial attenuation.

### 2.5. Confirmation of the Absence of Surface O-PS in the Natural Rough B. melitensis Rev.1 71036 and 44457 Vaccine Strains

**Figure 4:**
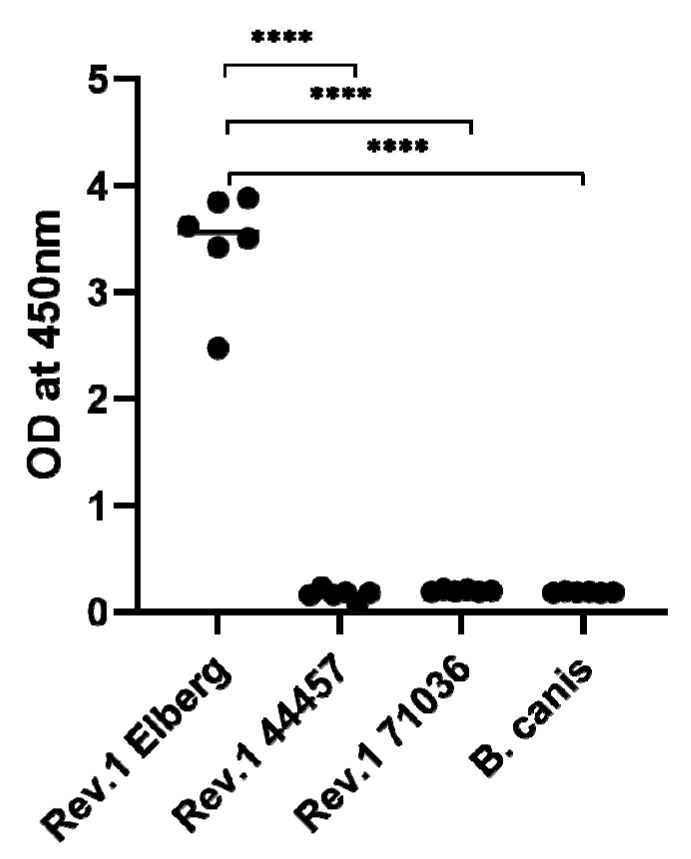
Whole cell immunoassay demonstrating the absence of surface O-polysaccharide (O-PS) in the natural rough *Brucella melitensis* Rev.1 71036 and 44457 vaccine strains. The evaluation of *Brucella* surface O-PS was determined using a purified Murine anti-*Brucella* O-PS Monoclonal antibody as described in Section 3. *B. melitensis* Rev.1 Elberg (smooth phenotype) was used as a positive control and *B. canis* (rough phenotype) as a negative control (**** = *p* < 0.0001, *t* test).

In order to determine the presence or absence of surface O-PS in the natural rough *B. melitensis* Rev.1 71036 and 44457 vaccine strains, an immunoassay was performed using a highly purified anti-O-PS Monoclonal antibody, as described in Section 3. Figure 4 clearly demonstrates the absence of surface O-PS in both rough strains, emphasizing the relevance of the specific detected gene mutations, associated to LPS biosynthesis, in the formation of the bacterial rough phenotype. 

## 3. Materials and Methods 

### 3.1. Strain Information, Extraction of the Genomic DNA, and Sequencing

*B. melitensis* Rev.1 and 16M genomes and annotations were downloaded from NCBI (accession numbers: NC_003317.1 and NC_003318.1 for 16M; CP024715 and CP024716 for Rev.1). Twenty-four smooth Rev.1 strains and two rough Rev.1 strains were used in this study (Supplementary Table S1). The phenotype of rough *Brucella* in suspension and on solid media was confirmed by acriflavine agglutination and crystal violet staining, respectively [48]. Total genomic DNA was extracted and purified using the DNeasy blood and tissue kit (Qiagen, Hilden, Germany) and was sent for sequencing in Illumina platforms at the Crown Institute for Genomics, G-INCPM, Weizmann Institute of Science, Israel. Illumina paired-end sequencing generated 1.2 million 2 X 250-bp reads (coverage, ~182 X).

### 3.2. SNP Detection

Following quality control with FastQC v0.11.5 [49], the reads were processed to trim adaptors and low-quality bases by using Trim Galore v0.5 [50]. Sequencing reads were then aligned to the Rev.1 reference genome using the Bowtie2 v2.3.4.1 program [51]. The SNPs were called using Samtools v1.7 [52]. Next, output SNPs were converted to VCF format and filtered (QUAL > 20) using BCFtools v1.7. Filtered SNPs were subjected to annotation by the SnpEff tool v4.3T [53]. The SnpEff database of the 16 M genome (2002 assembly; [54] and the Rev.1 genome (2018 assembly; [55,56] were built based on GenBank files that were downloaded from NCBI. To validate the detected SNPs and to detect additional mutations that were missed, the analysis was repeated using alignment against the 16M reference genome followed by SNP calling. In our analysis, we defined rough-specific mutations as variations exclusively appearing in Rev.1 rough strains and not in Rev.1 smooth strains.

### 3.3. Validation by Sanger Sequencing

Candidate genes containing rough-specific mutations were selected. PCR and sequencing primers were generated using Primer3 [57], and their specificity was tested using in-silico PCR [58] against the Brucella reference genome. Amplicons were sequenced on an Applied Biosystems 3730 XL DNA Analyzer. Sequenced reads were aligned to the reference genes and mutations were viewed in the Serial Cloner tool [59]. Multiple sequence alignment was done with T-Coffee [60] and visualized with the Boxshade tool [61].

### 3.4. Structural Analysis

#### 3.4.1. Motif Pattern and Domain Identification

Motifs and patterns in the analyzed proteins were found using Prosite at https://prosite.expasy.org/prosite.html [62]. Domain identification was done using PFAM, and can be found at https://pfam.xfam.org/ [63].

#### 3.4.2. 3D Protein Structure Analysis

For BMEII0899, the crystal structure of its paralog phosphoglucomutase was used (PDB ID 4HJH, article is unpublished). For BMEII0256 (glutathione S-transferase family protein), BMEII0418 (magnesium transporter), BMEI0267 (major facilitator superfamily (MFS) efflux pump) and BMEII0323 (NAD(P) transhydrogenase subunit alpha), no crystal structure was available and thus homology models were generated as follows. Template identification was done using Swissmodel [64] and HHPRED [65], followed by model generation using Modeller [66]. Default parameters were used for the template identification and homology modeling. The templates used for the model construction were BMEII0256—glutathione S-transferase from *Sinorhizobium meliloti* (PDB ID 4MDC, article is unpublished), BMEII0418—magnesium transporter MgtE protein from *Thermus thermophilus* (PDB ID 2ZY9, [30]), BMEI0267—*Escherichia coli* multidrug transporter MdfA (PDB ID 4ZP0, [41]) and BMEII0323—Alpha1 dimer of *T. thermophilus* transhydrogenase (PDB ID 4IZH, article is unpublished). Protein structures were visualized and images generated using Discovery Studio 4.5 Visualizer (BIOVIA, Dassault Systèmes, Discovery Studio 4.5 Visualizer, San Diego, CA, USA).

#### 3.4.3. Multiple Sequence Alignment

Multiple sequence alignment, was carried out as follows. Sequences were extracted using BLASTP (https://blast.ncbi.nlm.nih.gov/Blast.cgi?PAGE=Proteins; [67], with the protein sequence of the mutation as the query. The database used was a non-redundant protein sequences database (nr), with all other parameters used as default and results filtered as needed. The extracted sequences were used as input for the MAFFT online server [68], which can be found at https://mafft.cbrc.jp/alignment/server/, using its default parameters. Results were viewed using MSAViewer, available from the MAFFT online server.

#### 3.4.4. Conservation Analysis

Conservation analysis was done using ConSurf server [69], a bioinformatics tool for estimating the evolutionary conservation of amino acid positions in a protein molecule based on the phylogenetic relations between homologous sequences, based on a residue’s structural and functional importance. The server can be found at https://consurf.tau.ac.il/, and was used with the protein crystal structure, or homology model that was generated, with default parameters.

#### 3.4.5. Acid and Oxidative Stress Assays

For the acid stress assay, 1 × 10^8^ CFU of bacteria were grown in 10 mL of either a pH 4.4 or a pH 7.4 TSB (tryptic soy broth) culture and incubated for 3 h at 37 °C. For the oxidative stress assay, 1 × 10^8^ CFU of bacteria were grown in 10 mL TSB culture containing 0 or 10 mM Hydrogen peroxide solution (H_2_O_2_; Sigma-Aldrich Rehovot, Israel) and incubated for 2 h at 37 °C. Serial dilutions of the culture were then plated on tryptic soy agar plates to enumerate the CFUs. All experiments were repeated three times, independently, in triplicate.

#### 3.4.6. Whole Cell Immunoassay for the Detection of Surface Brucella OPS

10^7^ CFU/mL bacteria were harvested, resuspended in 0.5% phenol (Sigma Aldrich Rehovot, Israel) saline and incubated for 2 days at 37 °C. After 2 days, deactivated bacteria were centrifuged at 4500 rpm for 10 min and dissolved in 5 mL of coating buffer as previously described [70]. Two-hundred microliters of the dissolved bacteria were then placed in wells of a 96 well ELISA plate (Thermo-Nunc Maxisorp), incubated overnight at 37 °C, washed thrice with PBS-Tween and blocked with 1.5% Bovine Serum Albumin (Sigma) for 2 h at room temperature. The plate was then washed thrice with PBS-Tween and 100 µL of purified anti-O-PS Murine Monoclonal antibody (SVANOVIR^®^ Brucella-Ab C-ELISA kit; 1:10) was added to each well and incubated for 2 h at 37 °C. The plate was washed again, incubated for 1 h at 37 °C with 100 µL of goat anti-mouse IgG HRP conjugate (Thermo Fisher Scientific, Waltham, MA, USA; 1:1200), and developed with TMB (Tetramethylbenzidine) substrate (TMB Substrate Kit, Thermo Fisher Scientific) for 5 min. To stop the reaction, 100 µL of 2 M H_2_SO_4_ (Sigma Aldrich) was added to each well and absorbance was measured at 450 nm in a BioTek microplate reader. The experiment was repeated twice, independently, in triplicate. All the work with *Brucella* strains was performed at a biosafety level 3 laboratory in the Kimron Veterinary Institute, Bet Dagan, Israel.

## 4. Conclusions

Through a comparative genomic analysis, we found several genes, in natural rough *B. melitensis* Rev.1 vaccine strains, that play key roles in bacterial LPS biosynthesis and virulence, which contain unique frameshift or missense mutations, as compared with smooth Rev.1 strains. We suggest that these genes are involved specifically in the molecular mechanisms underlying the formation of the *B. melitensis* Rev.1 rough phenotype, and contribute to the attenuation of the rough versus smooth strains. Further characterization, via mutation and knockout experiments, is required to conclusively determine the role of these genes in affecting *Brucella* morphology and virulence attenuation of the *B. melitensis* Rev.1 vaccine strain. 

## Supplementary Materials

The following are available online at https://www.mdpi.com/1422-0067/21/24/9341/s1. Figure S1: Missense mutation within NAD(P) transhydrogenase subunit alpha protein, Figure S2: Missense mutation within Magnesium transporter, Figure S3: Missense mutation within the MFS transporter protein, Figure S4: In vitro stress survival of the natural rough B. melitensis Rev.1 71036 and 44457 vaccine strains, Table S1: List of Bacterial strains used for this study, Table S2: List of genomic SNPs.
