# Supplementary material for: Genomic Analysis of Natural Rough Brucella melitensis Rev.1 Vaccine Strains: Identification and Characterization of Mutations in Key Genes Associated with Bacterial LPS Biosynthesis and Virulence
Source: Int J Mol Sci. 2020 Dec 8;21(24):9341. doi: 10.3390/ijms21249341 (PMC7762576; doi:10.3390/ijms21249341)
Supplement: Supplementary file 1 [file ijms-21-09341-s001.zip › Supplementary Figures new.pptx]

## Slide 1
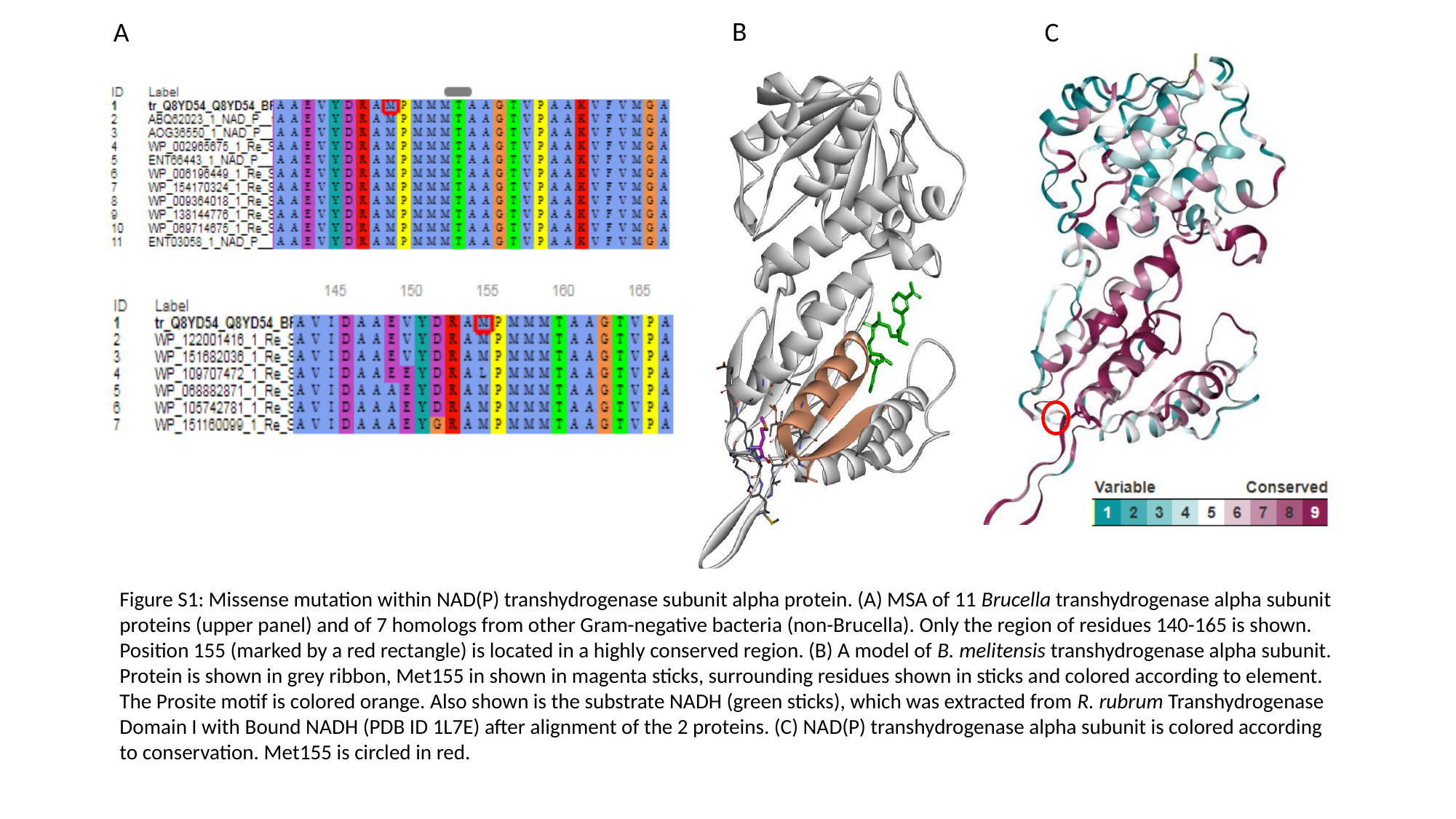

B
A
C
Figure S1: Missense mutation within NAD(P) transhydrogenase subunit alpha protein. (A) MSA of 11 Brucella transhydrogenase alpha subunit proteins (upper panel) and of 7 homologs from other Gram-negative bacteria (non-Brucella). Only the region of residues 140-165 is shown. Position 155 (marked by a red rectangle) is located in a highly conserved region. (B) A model of B. melitensis transhydrogenase alpha subunit. Protein is shown in grey ribbon, Met155 in shown in magenta sticks, surrounding residues shown in sticks and colored according to element. The Prosite motif is colored orange. Also shown is the substrate NADH (green sticks), which was extracted from R. rubrum Transhydrogenase Domain I with Bound NADH (PDB ID 1L7E) after alignment of the 2 proteins. (C) NAD(P) transhydrogenase alpha subunit is colored according to conservation. Met155 is circled in red.

## Slide 2
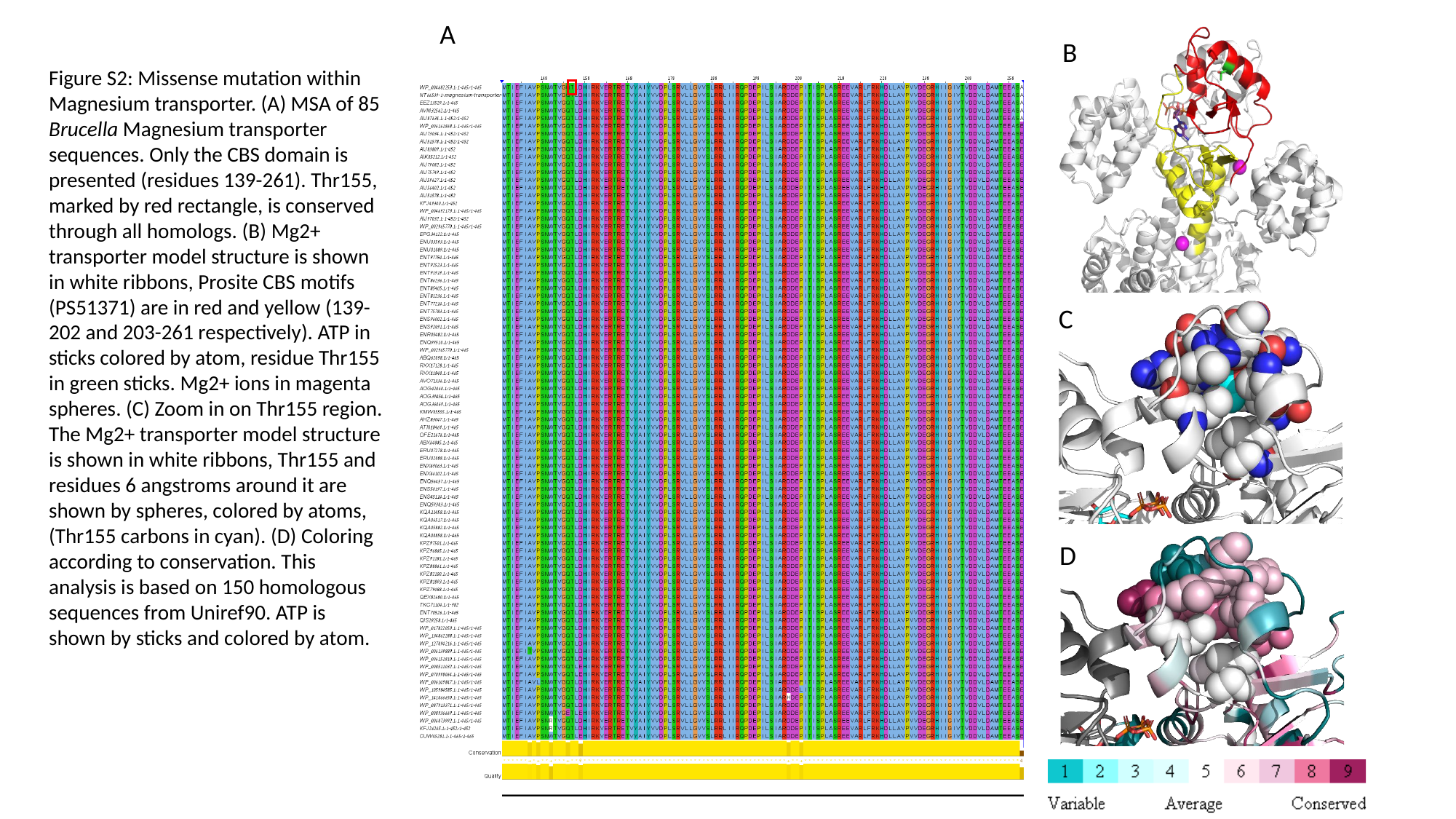

A
B
Figure S2: Missense mutation within Magnesium transporter. (A) MSA of 85 Brucella Magnesium transporter sequences. Only the CBS domain is presented (residues 139-261). Thr155, marked by red rectangle, is conserved through all homologs. (B) Mg2+ transporter model structure is shown in white ribbons, Prosite CBS motifs (PS51371) are in red and yellow (139-202 and 203-261 respectively). ATP in sticks colored by atom, residue Thr155 in green sticks. Mg2+ ions in magenta spheres. (C) Zoom in on Thr155 region. The Mg2+ transporter model structure is shown in white ribbons, Thr155 and residues 6 angstroms around it are shown by spheres, colored by atoms, (Thr155 carbons in cyan). (D) Coloring according to conservation. This analysis is based on 150 homologous sequences from Uniref90. ATP is shown by sticks and colored by atom.
C
D

## Slide 3
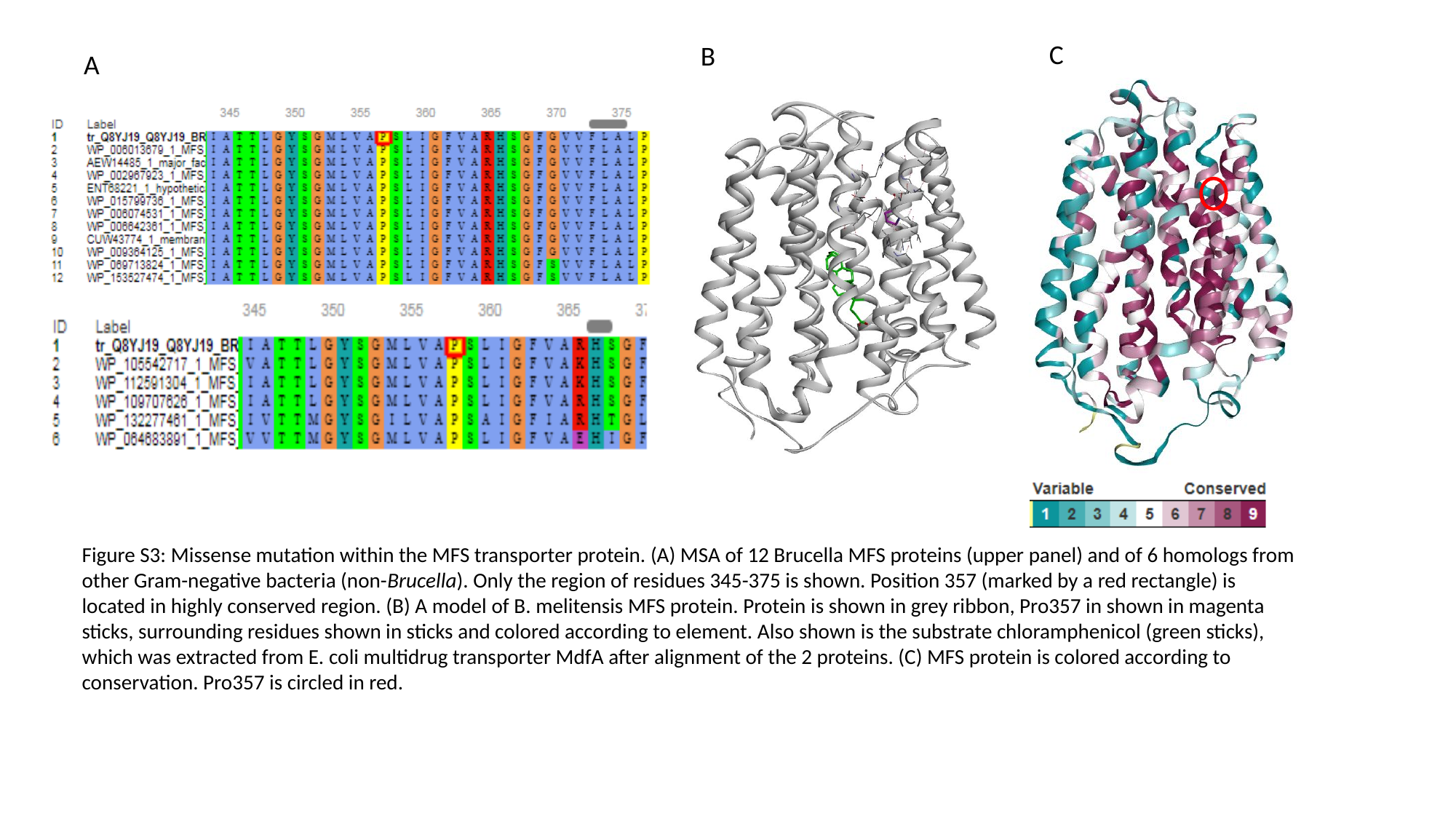

C
B
A
Figure S3: Missense mutation within the MFS transporter protein. (A) MSA of 12 Brucella MFS proteins (upper panel) and of 6 homologs from other Gram-negative bacteria (non-Brucella). Only the region of residues 345-375 is shown. Position 357 (marked by a red rectangle) is located in highly conserved region. (B) A model of B. melitensis MFS protein. Protein is shown in grey ribbon, Pro357 in shown in magenta sticks, surrounding residues shown in sticks and colored according to element. Also shown is the substrate chloramphenicol (green sticks), which was extracted from E. coli multidrug transporter MdfA after alignment of the 2 proteins. (C) MFS protein is colored according to conservation. Pro357 is circled in red.

## Slide 4
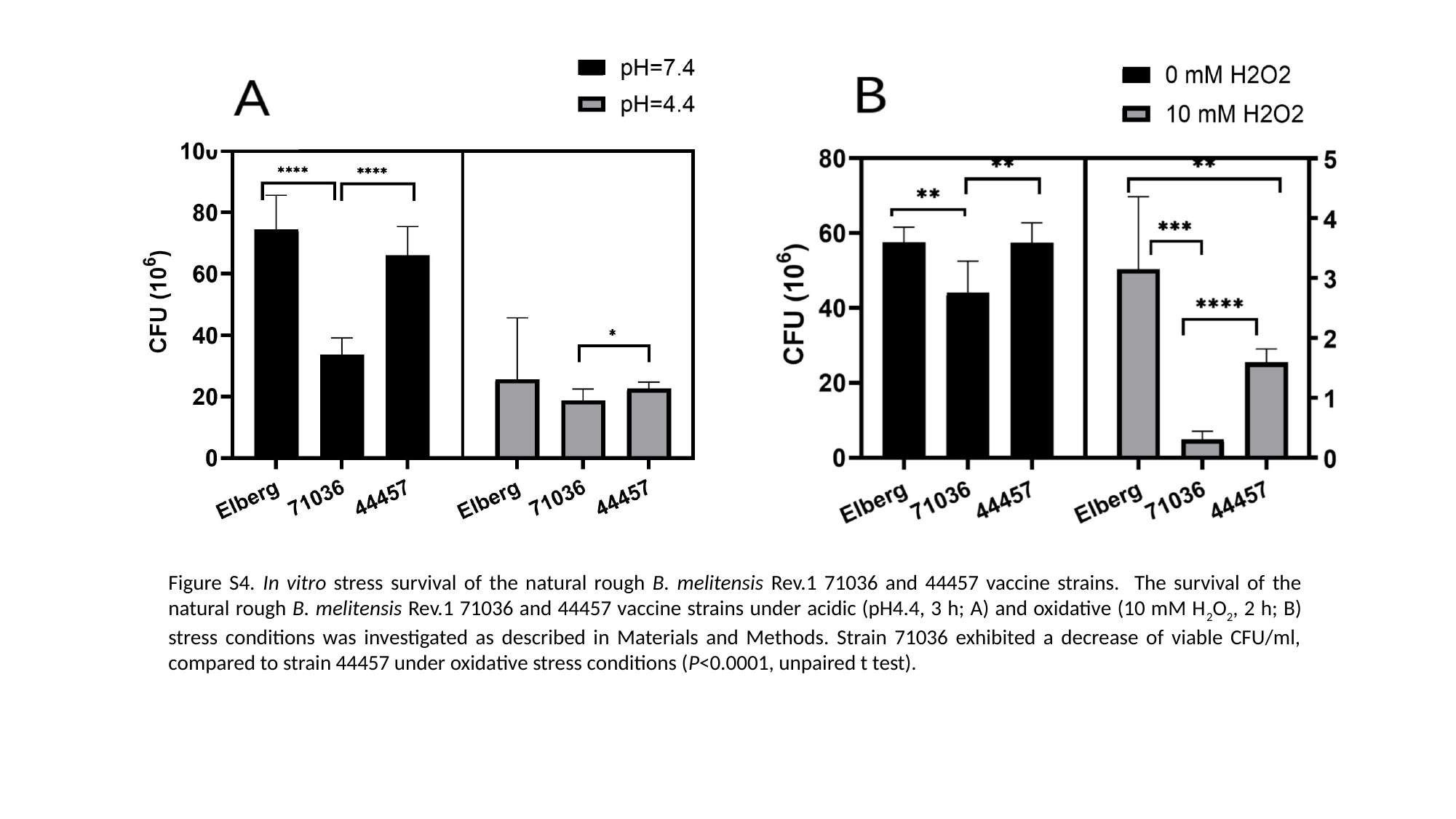

Figure S4. In vitro stress survival of the natural rough B. melitensis Rev.1 71036 and 44457 vaccine strains. The survival of the natural rough B. melitensis Rev.1 71036 and 44457 vaccine strains under acidic (pH4.4, 3 h; A) and oxidative (10 mM H2O2, 2 h; B) stress conditions was investigated as described in Materials and Methods. Strain 71036 exhibited a decrease of viable CFU/ml, compared to strain 44457 under oxidative stress conditions (P<0.0001, unpaired t test).
